# Association of Electronic Prescribing of Controlled Substances With Opioid Prescribing Rates

**DOI:** 10.1001/jamanetworkopen.2020.27951

**Published:** 2020-12-21

**Authors:** Jordan Everson, Audrey K. Cheng, Stephen W. Patrick, Stacie B. Dusetzina

**Affiliations:** 1 Department of Health Policy, Vanderbilt University School of Medicine, Nashville, Tennessee; 2 Department of Biomedical Informatics, Vanderbilt University School of Medicine, Nashville, Tennessee

## Abstract

**Importance:**

The majority of US states have passed mandates requiring the use of electronic prescribing of controlled substances (EPCS) as a tool to reduce rates of opioid prescribing. It is not known whether increasing use of EPCS will have the intended effect.

**Objective:**

To assess the association between use of EPCS and trends in opioid prescribing.

**Design, Setting, and Participants:**

In this retrospective, longitudinal cohort study of all patients and prescribers in the 50 US states and the District of Columbia from 2010 to 2018, changes in state-level use of EPCS and concurrent changes in opioid prescribing in each state are described. Then the association between changes in the use of EPCS and opioid prescribing are estimated using state and year fixed-effects models that include covariates for policy change and state demographic change. Data Analysis was performed on May 5, 2020.

**Main Outcomes and Measures:**

The proportion of controlled substances in each state prescribed using EPCS based on opioid prescriptions per 100 persons and morphine milligram equivalents (MME) of opioids.

**Results:**

In 2018, the population-weighted percent of opioids prescribed using EPCS was 27%, up from 0% as of 2013. National rates of opioid prescriptions decreased from 78 prescriptions per 100 persons in 2013 to 53 in 2018. Over the same period, there was a decrease from 64 071 MME per 100 persons in 2013 to 40 906 MME per 100 persons in 2018, representing 36% of the 2013 level. By 2018, EPCS increased to 69.4% in states with mandates for its use and 23.6% in states without mandates. In multivariable models, a 10 percentage-point increase in the use of EPCS was associated with an additional 2 prescriptions per 100 persons (95% CI, 1.3-2.8) and a 0.8% (95% CI, 0.06%-1.5%) increase in MME per 100 persons.

**Conclusions and Relevance:**

These data suggest that an increased use of EPCS was not associated with decreased opioid prescribing or a decrease in the amount prescribed and may have been associated with a small increase in opioid prescribing. Opioid prescribing is associated with a variety of social and public health factors, and thus, despite the appeal, EPCS adoption alone may be insufficient to reduce opioid prescribing. Policy makers should consider levers to ensure that EPCS is integrated with outside data and that information is actively used to inform prescribing decisions.

## Key Points

**Question:**

Have states with greater increases in electronic prescribing of controlled substances (EPCS), which is intended to reduce opioid prescribing rates by reducing fraud and facilitating decision support, experienced greater reductions in opioid prescribing?

**Findings:**

In this longitudinal analysis of Surescripts reports on the use of EPCS and Center of Disease Prevention and Control opioid prescribing rate maps, increasing the use of EPCS by 10 percentage points was associated with 2 more opioid prescriptions per 100 persons and 0.8% more morphine milligram equivalents.

**Meaning:**

The EPCS has not been associated with reductions in opioid prescribing rates, and achieving benefit may require greater integration and effective presentation of data.

## Teaser

This retrospective, longitudinal cohort study assesses the association between use of electronic prescribing of controlled substances and trends in opioid prescribing.

## Introduction

Prescription opioids are an important component of the opioid epidemic, contributing to 55% of the approximately 400 000 opioid-related overdose deaths that occurred between 1999 and 2017.^1,2^ Recently, electronic prescribing of controlled substances (EPCS) has been pursued as a means to “[decrease] rates of prescription opioid addiction, abuse, diversion, and death by making it more difficult to ‘doctor-shop’ and alter prescriptions.”^3^^(p156)^ By February 2020, mandates for EPCS were in effect in 13 states and passed in an additional 14 states. In addition, Congress mandated that all controlled substances covered under Medicare Part D be prescribed using electronic prescribing by 2021.^4,5^ Yet despite wide policy support for EPCS, the association between the use of EPCS and opioid prescribing is unknown.

Enthusiasm for EPCS is due to its potential to detect and prevent opioid diversion by eliminating paper prescriptions,^6^ enabling cross-referencing of prescription drug monitoring program (PDMP) databases^7^ and facilitating electronic decision support tools.^8,9,10^ Based on these potential advantages, the implementation and use of EPCS may curb “doctor shopping,” prescription fraud, and overprescribing, thus leading to improved opioid outcomes. However, these potential benefits may not be realized if EPCS systems do not effectively display the information needed to identify prior prescriptions or evidence of fraud in a way that is useful to clinicians at the point of prescribing. Furthermore, EPCS may result in more prescribing by making it easier to electronically order controlled substances in a workflow similar to other prescriptions. Like other sources of electronic clinical decision support, the success of EPCS is not assured and may depend on the crucial factors of its implementation.^11,12^

Existing literature on EPCS is limited. Previous investigations have examined the challenges of adopting and impementing EPCS^13,14^; 1 cross-sectional study showed that hospitals with EPCS were not associated lower opioid prescribing in the surrounding county,^15^ and 1 single-site study showed that opioid-prescribing by emergency physicians dcreasedd after the introduction of EPCS mandates in New York but did not compare these trends with groups not using EPCS—a serious limitation given widespread decreases in opioid prescribing over the time period.^3^ In this study, we aim to build on the existing research by analyzing the association of the adoption of EPCS with opioid prescribing across the United States. We specifically examine trends in the adoption and use of EPCS and 2 measures of opioid prescribing across the United States from 2010 to 2018. In doing so, we aim to provide policy makers, prescribers, and patients with evidence of the association of the use of EPCS with the opioid epidemic.

## Methods

### Data

We compiled data from multiple publicly available sources to construct our analytic data set. We used data from annual reports published by Surescripts, a near-monopoly supplier of electronic prescribing,^16^ to measure the proportion of controlled substances prescribed using EPCS (hereafter referred to as “EPCS use”) by state.^17^ Owing to their high market share, Surescripts’ data on EPCS is likely to represent almost all use of EPCS. This measure was our primary independent variable. Using publicly available data,^18,19^ we categorized states based on whether they had a mandate for EPCS in effect, had passed a mandate but it was not yet in effect, or had not yet taken legal action toward a mandate as of the end of the study period. We reviewed the Strengthening the Reporting of Observational Studies in Epidemiology (STROBE) reporting guideline in reporting observational results. Because we used only aggregate, deidentified data, this works was not considered human subjects research and did not require approval by the institutional review board of Vanderbilt University.

We used 2010-2018 data related to the amount of opioids prescribed from 2 sources. Data on overall opioid prescriptions per 100 persons came from Centers for Disease Control and Prevention opioid prescribing rate Maps.^20^ We also used data from the US Drug Enforcement Administration’s Automation of Reports and Consolidated Orders System^21^ to measure the morphine milligram equivalents (MME) of opioids based on 8 prescription opioids used to relieve pain (codeine, fentanyl, hydrocodone, hydromorphone, meriperidine, morphine, oxycodone, and oxymorphone).^22,23,24^ We converted the weight of each opioid to MME using previously implemented approaches.^22,23,24^ The annual rate of opioid prescriptions per 100 persons and the relative change in MME per 100 persons since 2013 served as our primary outcome variables.

In multivariable analyses, we included additional data on state unemployment rates and poverty rates from single-year estimates of the American Community Survey because demographic factors have been shown to be associated with opioid use and may also be associated with the ability to invest in EPCS.^25,26^ In addition, we included the status of 4 related opioid policies, which all aimed at reducing opioid overuse and were likely to correlate to EPCS mandates and use, from the Prescription Drug Abuse Policy System: (1) prescription drug monitoring programs, (2) pain management clinic laws, (3) direct dispensing of controlled substances laws, and (4) opioid prescribing guidelines for acute emergency care.^27,28,29,30^

### Statistical Analysis

We examined population-weighted trends in EPCS use, opioid prescriptions, and percent change in MME of opioids across the United States since 2010, when EPCS was first legalized through amendment of the Controlled Substance Act. We then categorized states by the status of EPCS mandates as of the end of the study period, January 1, 2019, and compared rates of EPCS use across these groups. Electronic prescribing of controlled substances was not supported by vendors until 2013 (and its use was therefore 0), data prior to 2013 provides trends in opioid prescribing by state, and we used 2013 as the baseline for all comparisons. We also plotted changes in opioid prescriptions, percent change in MME of opioids, and EPCS use by state from 2013 to 2018. The relative size of change in each variable by state and correlation across variables indicates whether states with larger increases in EPCS use also saw larger decreases in opioid prescriptions.

Next, we estimated 2-way fixed-effects models to measure the adjusted association between increasing rates of EPCS use, percent change in MME of opioids relative to the national average in 2013, and opioid prescriptions per 100 persons. By including state and year fixed effects, these models account for time-invariant state-level factors and overall trends, respectively. Inclusion of demographic variables and the status of other opioid policies further accounts for key time-varying changes that might bias our estimates. In each model, we used analytic weights to account for differing populations of each state and heteroskedastic robust standard errors.

To examine the robustness of our approach, we also examined change in opioid prescribing and MME in each state relative to the rate in that state in 2013. In our primary model, states with high levels of opioid prescribing in 2013 that then, for example, reduced opioid prescribing by 10% of their 2013 level would receive more credit than states with low initial levels that similarly decreased by 10%. In contrast, this approach would give equal credit to both states that reduced rates in their state by 10%.

We also examined whether EPCS was more beneficial (ie, associated with decreased opioid prescribing) in states with high levels of opioid use in the baseline period, which might have more room to improve, or in states with low baseline rates of opioid prescribing, where the challenge may be more tractable. To do so, we divided states into those above and below the median rate of opioid prescribing in 2013 and reran both regression models.

Finally, we performed 2 additional robustness tests. First, to identify whether EPCS use was associated with lower doses when prescribed, we replicated our initial regression models using MME per prescription as the outcome variable We generated this variable by dividing the measure of MME per 100 persons by prescription per 100 persons. Second, to examine whether the association of EPCS use was greater in states with more robust PDMP laws, we replicated our initial models, including an interaction term between laws mandating that prescribers check PDMPs before prescribing and EPCS use. More details on these methods and results are available in the eAppendix, eFigure, and eTables 1, 2, and 3 in the Supplement.

All analyses were conducted in STATA MP 16 using the xtreg command and analytic weights (StataCorp), and visualizations were created in R 3.6 using ggplot2. Results were considered statistically significant if *P* < .05 in 2-sided tests.

## Results

**Figure 1.**
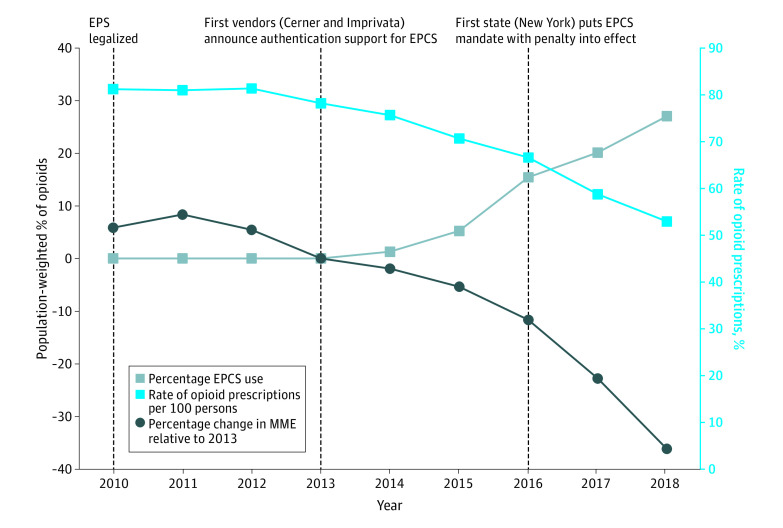
Trends in Opioid Prescribing, Morphine Milligram Equivalents (MME), and Electronic Prescribing of Controlled Substances (EPCS) Capability, 2010-2018

Our data included observations on 50 states and the District of Columbia over 9 years, yielding a total of 459 observations. In 2018, the population-weighted percent of opioids prescribed using EPCS was 27%, up from 0% as of 2013. Meanwhile, national rates of opioid prescriptions have decreased from 78 prescriptions per 100 persons in 2013 to 53 in 2018. Over the same period, there was a decrease from 64 071 MME per 100 persons in 2013 to 40 906 MME per 100 persons in 2018, representing 36% of the 2013 level (Figure 1).

**Figure 2.**
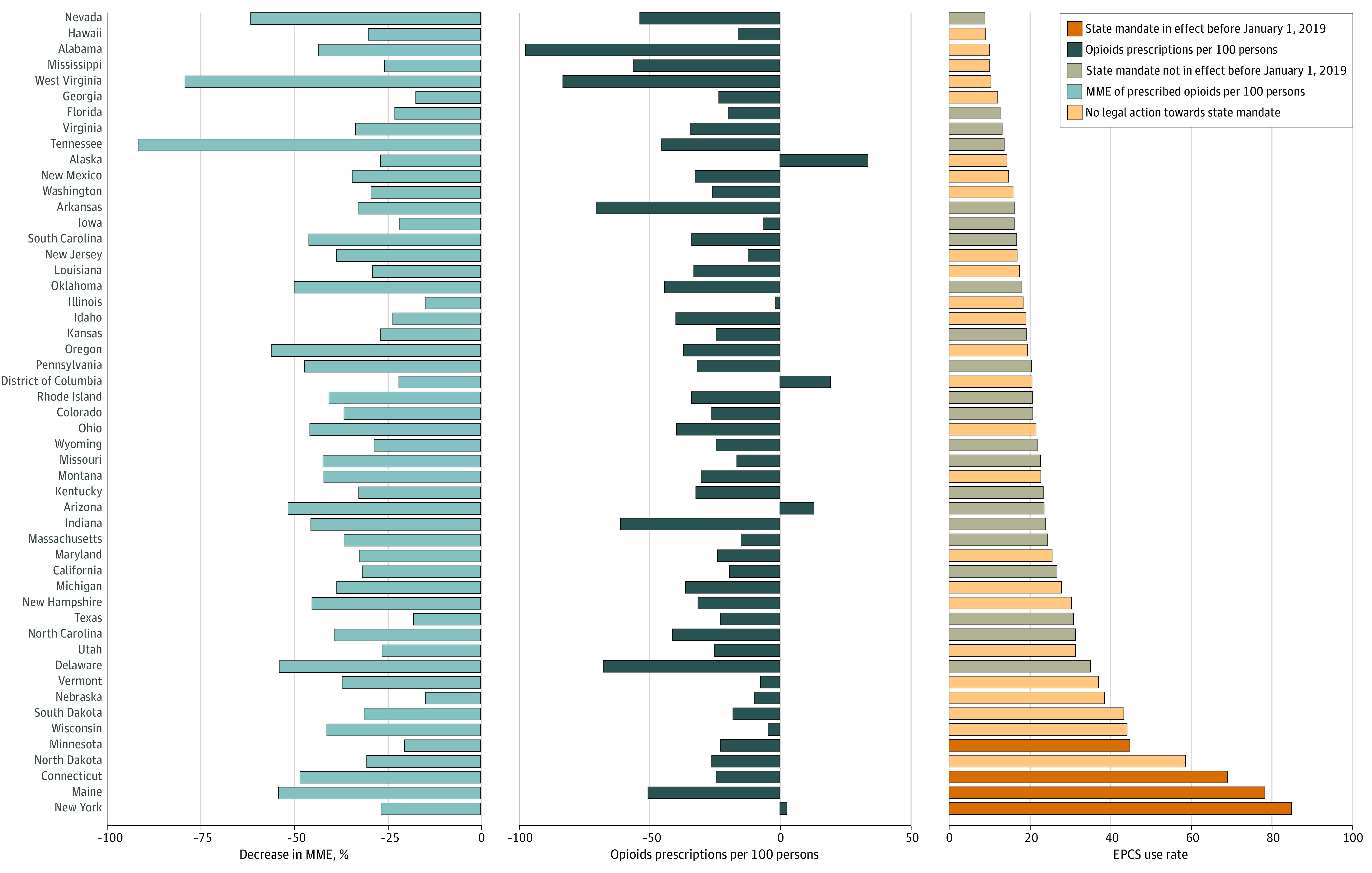
Change in Opioid Prescribing, Percent Morphine Milligram Equivalents (MME) of Prescribed Opioids, and Prescriber Electronic Prescribing of Controlled Substances (EPCS) Use, 2013-2018

EPCS use varied substantially by state (Figure 2), from 85% EPCS use in New York to 9% EPCS use in Nevada in 2018. Change in EPCS use from 2013 to 2018 was higher in states that had mandates for use prior to 2019 (mean increase in opioids prescribed using EPCS, 69.4%) in either states that had passed mandates but not yet put them in place by 2019 (21.0%) and states without any action toward mandates (23.6%).

Opioid prescriptions decreased nationally by a mean of 28.7 (95% CI, 22%-35%) opioid prescriptions per 100 persons but varied by state, with the largest decrease of 98 opioid prescriptions per 100 persons (117% of the national average) in Alabama and an increase of 34 prescriptions per 100 persons in Alaska (41% of the national average) between 2013 and 2018. There was a small, positive association between the change in EPCS use and the change in opioid prescriptions over this period (Spearman rank correlation = 0.26), meaning that states with higher EPCS use had smaller decreases in opioid use. The mean decrease in MME across states was 25 299 (37.1% of the national average) and ranged from decreases of 62 407 MME per 100 persons in Tennessee (91.5% of the national average) to 10 087 in Nebraska (14.8% of the national average). The Spearman rank correlation between change in EPCS use and change in MME was −0.002.

When considering relative changes in opioid prescribing by state, opioid prescriptions per 100 persons decreased by 31% (95% CI, 24%-38%), and this varied from a decrease of 73% of the 2013 baseline in Delaware to a 53% increase in Alaska (eFigure in the Supplement). MME per 100 persons decreased by 37% (95% CI, 34%-39%) of the state’s 2013 baseline, ranging from 54% of the 2013 baseline in West Virginia to just 20% of the baseline in Georgia.

In multivariable regression models, we observed that a 10-percentage-point increase in EPCS use was associated with an increase in opioid prescriptions of 2.0 prescriptions per 100 persons (95% CI, 1.3-2.8), which represents a 2.5% increase from the mean levels in 2013 (Table 1). The same increase in EPCS use was associated with a small increase in MME of 0.8% (95% CI, 0.06%-1.5%) from the mean level in 2013, which is equivalent to 565.9 MME (95% CI, 41.8-1090.0). We did not observe an association between EPCS and lower opioid prescribing (ie, a beneficial association) in models that adjusted the outcome variable by the states’ baseline rates and observed an association between EPCS use and greater opioid prescribing in 1 model (eTable 1 in the Supplement).

**Table 1.** Multivariable Analysis of Association Between EPCS Use and Prescription Opioids^a^

Parameter	Regression coefficient (SE)	
	Opioid prescriptions per 100 persons	% Change in MME per 100 persons (relative to 2013)
Rate of EPCS use (10% increments)	2.058 (0.374)^b^	0.83 (0.383)
Unemployment rate	0.526 (0.730)	5.266 (2.565)^c^
Poverty rate	−1.768 (1.193)	−3.958 (1.525)^c^
PDMP implemented	1.422 (2.277)	−11.775 (10.538)
Pain management clinic law	−2.583 (4.360)	1.717 (7.692)
Direct dispensing law	0.471 (1.355)	0.311 (2.499)
ED prescribing guidelines	−2.117 (2.067)	−0.893 (3.132)
Constant	102.605 (16.979)^b^	107.938 (24.320)^b^
No. of observations	459	459
No. of states	51	51

Abbreviations: ED, emergency department; EPCS, electronic prescribing of controlled substances; MME, milligram morphine equivalent; PDMP, prescription drug monitoring program.

^a^ State and year fixed effects were included in each regression.

^b^ *P* < .01, generated using robust standard errors.

^c^ *P* < .05, generated using robust standard errors.

When we divided states into groups that had high and low baseline levels of opioid prescribing, a 10-percentage-point increase in EPCS use was associated with 1.1 (95% CI, 0.46-1.8) more opioids per 100 persons in states with low baseline opioid prescribing rates, with no association in states with high baseline opioid prescribing rates or for changes in MME (Table 2). In general, standard errors were substantially larger in states with high baseline rates of opioid prescribing (eTable 2 in the Supplement).

**Table 2.** Multivariable Analysis of Association Between EPCS Use and Prescription Opioids Stratified by National Average^a^

Parameter	Regression coefficient (SE)			
	Opioid prescriptions per 100 persons, No.		% Change in MME per 100 persons (relative to 2013)	
	Under median in 2013	Over median in 2013	Under median in 2013	Over median in 2013
EPCS use rate (10% increments)	1.317 (2.673)	1.120 (0.321)^b^	0.421 (4.145)	0.001 (0.419)
Unemployment rate	0.177 (0.866)	−0.081 (0.922)	2.137 (1.777)	7.319 (3.601)
Poverty rate	−2.727 (1.618)	0.418 (1.076)	−4.673 (2.326)	−1.618 (1.593)
PDMP implemented	7.366 (2.760)^c^	−3.485 (1.951)	6.143 (5.466)	−19.912 (12.531)
Pain management clinic law	−8.484 (2.404)^b^	12.545 (8.567)	−4.851 (5.060)	−6.617 (7.227)
Direct dispensing law	1.822 (2.112)	1.620 (1.267)	3.880 (3.502)	0.856 (3.114)
ED prescribing guidelines	−2.837 (2.942)	−1.778 (1.694)	−1.086 (5.631)	0.655 (3.388)
Constant	142.815 (26.614)^a^	60.912 (13.117)^a^	164.092 (38.660)^a^	46.189 (37.226)
No. of observations	225	234	225	234
*R*^2^ value	0.861	0.800	0.786	0.694
No. of states	25	26	25	26

Abbreviations: ED, emergency department; EPCS, electronic prescribing of controlled substances; MME, milligram morphine equivalent; PDMP, prescription drug monitoring program.

^a^ State and year fixed effects were included in each regression.

^b^ *P* < .01, generated using robust standard errors.

^c^ *P* < .05, generated using robust standard errors.

In the robustness check examining whether EPCS use was associated with MME per prescription (rather than per person), we observed that greater EPCS use was associated with lower MME per prescription, such that a 10-percentage-point increase in EPCS use was associated with a decrease of 19 MME (95% CI, 12-27 MME) from a baseline of 838 in 2013 (eTable 2 in the Supplement). In the final robustness check, we did not observe a statistically significant association between mandatory PDMP checking and EPCS use when estimating opioid prescriptions per 100 persons. However, we did observe an association between mandatory PDMP checking and EPCS use when estimating MME per person, such that states without mandatory PDMP checking observed an increase in MME per person, while states with mandatory PDMP checking did not (eTable 3 in the Supplement).

## Discussion

In this study, we used national data to evaluate the association between EPCS use and opioid prescribing. Despite enthusiasm for EPCS,^7^ these data suggest that its increased use was not associated with decreased opioid prescribing or a decrease in the amount of opioids prescribed, and it may have been associated with a small increase in opioid prescribing rates.

Complex social and public health factors may be associated with the high levels of opioid prescribing, and multifaceted solutions are likely needed to reduce those levels. EPCS can play a role in reducing opioid prescribing by addressing 2 key needs: ensure that the prescribing system is secured against fraud and abuse and that prescribers are fully informed about the other opioids their patients take.

On its own, EPCS’s primary benefits may be to make it harder to alter paper prescriptions or otherwise commit fraud. But this type of fraud is likely a small part of overall prescribing rates, so that this function alone may have a limited effect. A second potential benefit of EPCS is to facilitate the use of default or recommended doses, which may explain our finding that EPCS use was associated with lower MME per prescription. Beyond these functions, EPCS itself may not directly dissuade prescribers from prescribing opioids. Instead, it may, in fact, make it simpler to place an order for a controlled substance because, relative to a paper-based workflow for prescribing controlled substances, EPCS is more similar to the electronic process used to write other prescriptions. This dynamic may have resulted in the small increase in prescribing rates we observed.

We saw some evidence that EPCS use was associated with greater MME per person in states without mandatory PDMP checking but was not associated with greater MME per person in states with mandated PDMP checking. This finding supports prior research that states with more robust PDMPs—including data integration—have seen more benefit.^31^ It also supports the potential for PDMP checking to alter how EPCS is associated with prescribing behavior but highlights that, to date, this association is not sufficiently beneficial to reduce prescribing. To provide maximum benefit, EPCS must facilitate the use of data from PDMPs and other external sources through interoperable data exchange and make it easier for clinicians to understand the data in a certain context. EPCS should simplify the process of providing accurate and usable data to PDMPs and showing PDMP data to prescribers through effective clinical decision support. Despite the intuitive appeal of robust information sharing and automated decision support, the literature on both topics indicates that achieving benefit from these technologies depends on the specific context of their implementation and use.^12,28,32,33^ Similarly, successfully reducing opioid prescribing through EPCS use likely depends on factors related its implementation.

Since 2015, public policy on EPCS has progressed rapidly, such that most states have passed laws mandating its use, and the federal government has mandated its use for Medicare Part D drugs. Our data indicate that EPCS use has not performed as expected and that mandates may be a necessary but insufficient step toward decreasing opioid prescribing. For policy makers, this points to a need to ensure that relevant incentives exist to use the data and that factors related to how EPCS is implemented facilitate its effective use.

Policy makers should consider approaches to pair the mandated use of EPCS with incentives to encourage effective review of opioid information. One option would be for the Center for Medicare & Medicaid Services to include EPCS use and integration with the PDMP as a metric in the Merit-Based Incentive Payment System and Advanced Payment Models. Like other measures in the Promoting Interoperability component of the Merit-Based Incentive Payment System, prescribers could be asked to report the proportion of patients with opioid prescriptions for whom EPCS was used and the proportion of patients for whom information from the PDMP was checked within the electronic prescribing workflow. Strengthening laws around EPCS to ease or mandate integration, and not just use, of PDMP data may lead to a greater impact from both technologies.

Beyond direct incentives, policy makers, health information technology- developers, and health care delivery system leaders should carefully consider how to ensure EPCS is maximally useful to clinicians. For instance, the Office of the National Coordinator for Health Information Technology could consider requiring and testing that all certified electronic health records are able to present data related to opioid prescriptions within health care professional workflows. At the state level, health information exchange organizations may be well positioned to serve as catalysts around learning how to best make use of external data because they pursue that goal in other contexts.

### Limitations

Our data focuses on mean values across states rather than the association between individual prescribers’ use of EPCS and prescribing. This level of analysis allows us to observe aggregate trends by state but does not allow for more detailed investigation. For instance, EPCS use could facilitate decision support and PDMP checking that could alleviate prescriber concerns over “doctor shopping” and make them more willing to prescribe opioids for legitimate pain, thereby serving as a countervailing force against the blunt national trend against opioid prescribing. Our data indicate that EPCS use is associated with changing patterns of opioid prescribing, but additional, focused research is needed to track these specific trends.

A second important limitation is that states, local governments, health systems, and other groups have pursued an array of approaches to combat the opioid epidemic and decrease the rate of opioid prescribing. Although our analytic approach accounts for time-invariant omitted variable bias, it does not account for unobserved changes in policy or practice across states that may be associated with EPCS use and opioid prescribing. We have included several measures related to the passage of important laws and economic trends potentially relevant to opioid prescriptions to account for these changes.

Finally, our regression modeling framework relies on a few assumptions that cannot be validated: that past levels of opioid prescribing do not influence EPCS use in later years, that past use of EPCS does not influence opioid prescribing in subsequent years, and that modeled associations are additive.^34^ Given the small apparent association between EPCS use and current opioid prescribing and the brief time period over which any given prescribing event takes place, there is reason to believe these assumptions are valid.

## Conclusions

Using national data on EPCS use since its legalization, our findings indicate that the potential benefits from EPCS use were not realized during the initial phase of its adoption. Nevertheless, it is likely to be an important component in helping clinicians make fully informed decisions about whether to prescribe opioids. The data indicate that mandating its use may be only a starting point in building informatics-based practices aimed at addressing the opioid epidemic.

## Notes

Supplement. **eAppendix.** Supplemental Material **eFigure.** Percent Change in Opioid Prescribing, Percent MME of Prescribed Opioids and Prescriber EPCS, 2013-2018 Utilization Relative to the State’s Baseline Rates in 2013 **eTable 1.** Multivariate Analysis of Relationship Between EPCS Use and Prescription Opioids Relative to State Baseline **eTable 2.** Multivariate Analysis of Relationship Between EPCS Use and Milligram Morphine Equivalent (MME) Prescription **eTable 3.** Multivariable Analysis of Relationship of Interaction Between EPCS Use, Mandated PDMP Use and Opioid Prescriptions
