# Supplementary material for: Association of Electronic Prescribing of Controlled Substances With Opioid Prescribing Rates
Source: JAMA Netw Open. 2020 Dec 21;3(12):e2027951. doi: 10.1001/jamanetworkopen.2020.27951 (PMC7753903; doi:10.1001/jamanetworkopen.2020.27951)
Supplement: Supplement. — eAppendix. Supplemental Material eFigure. Percent Change in Opioid Prescribing, Percent MME of Prescribed Opioids and Prescriber EPCS, 2013-2018 Utilization Relative to the State’s Baseline Rates in 2013 eTable 1. Multivariate Analysis of Relationship Between EPCS Use and Prescription Opioids Relative to State Baseline eTable 2. Multivariate Analysis of Relationship Between EPCS Use and Milligram Morphine Equivalent (MME) Prescription eTable 3. Multivariable Analysis of Relationship of Interaction Between EPCS Use, Mandated PDMP Use and Opioid Prescriptions [file jamanetwopen-e2027951-s001.pdf]

Supplemental Online Content

Everson J, Cheng AK, Patrick SW, Dusetzina SB. Association of electronic prescribing of controlled substances with opioid prescribing rates. *JAMA Netw Open*. 2020;3(12):e2027951. doi:10.1001/jamanetworkopen.2020.27951

eAppendix. Supplemental Material

eFigure. Percent Change in Opioid Prescribing, Percent MME of Prescribed Opioids and Prescriber EPCS, 2013-2018 Utilization Relative to the State's Baseline Rates in 2013

eTable 1. Multivariate Analysis of Relationship Between EPCS Use and Prescription Opioids Relative to State Baseline

eTable 2. Multivariate Analysis of Relationship Between EPCS Use and Milligram Morphine Equivalent (MME) Prescription

eTable 3. Multivariable Analysis of Relationship of Interaction Between EPCS Use, Mandated PDMP Use and Opioid Prescriptions

This supplemental material has been provided by the authors to give readers additional information about their work.

© 2020 Everson J et al. *JAMA Network Open.*

eAppendix. Supplemental Material

We performed a variety of robustness checks to validate the results of our primary models examing the relationship between the use of electronic prescribing of controlled substances and opioid prescribing rates.

In the first robustness check, we recalculated the primary dependent variables so that the denominator was state-specific rates of opioid prescribing in the first year of EPCS use (2013) rather than national rates. This approach reduces potential bias if states with high or lower rates of opioid prescribing in 2013 were more likely to begin using EPCS. Our results, shown on eTable 1, were consistent in these models, which showed an increase in opioid prescriptions per 100 persons given greater EPCS use but no relationship between EPCS use and milligrams morphine equivalent (MME) per person.

In the second robustness check, we examined changes in MME per prescription (rather than per person). On eTable 2, we show that a 10 percentage point increase in EPCS use was associated with reduced MME per prescription of about 19 MME (95% CI: -27 MME - -12 MME) from a baseline rate of 838 in 2013 (Column 1). Similarly, we observed a 2.0 percent (95% CI: -3.0 percent - -1.0 percent) decrease in average state-level MME per prescription (Column 2).

In the third robustness check, we investigated whether EPCS Use was more effective at reducing opioid prescribing in States that had mandated PDMP checking. To do so, we identified the year that states mandated PDMP checking from multiple sources[1,2,3] and created a variable indicating whether mandatory PDMP check was in place in a given state in a given year. We then interacted this variable with our measure of EPCS use. In the model predicting opioid prescriptions per 100 persons (eTable 3, Column 1), the interaction between EPCS use and mandatory PDMP checking was not statistically significant, such that in both states with and without mandatory PDMP checking, greater EPCS use was associated with a greater number of opioid prescriptions per 100 persons. Specifically, a 10 percentage point increase in EPCS use was associated with 2.4 more prescriptions per 100 persons in States without PDMP checking (95% CI: 0.5-4.3) and 2.0 more prescriptions per 100 persons in States with PDMP checking (95% CI: 1.3 – 2.8). In the model predicting MME per 100 persons, the interaction between EPCS use and mandatory PDMP checking was statistically significant such that a 10 percentage point increase in EPCS use was associated with 4.4 more MME per 100 persons in States without mandatory PDMP checking ((%% CI: 1.0 MME – 7.7 MME) and was not associated with greater MME in States with mandatory PDMP checking (0.6 MME; 95% CI: -0.15 MME – 1.3 MME).

---

[1] Prescription Drug Monitoring Program Training and Technical Assistance Center. https://www.pdmpassist.org/State

[2] Buchmueller, Thomas C., and Colleen Carey. "The effect of prescription drug monitoring programs on opioid utilization in Medicare." *American Economic Journal: Economic Policy* 10.1 (2018): 77-112.

[3] We performed an online search via Google to confirm dates for three States: Delaware, Hawaii and the District of Columbia.

© 2020 Everson J et al. *JAMA Network Open.*

eFigure 1. Percent Change in Opioid Prescribing, Percent MME of Prescribed Opioids and Prescriber EPCS, 2013-2018 Utilization Relative to the State's Baseline Rates in 2013.

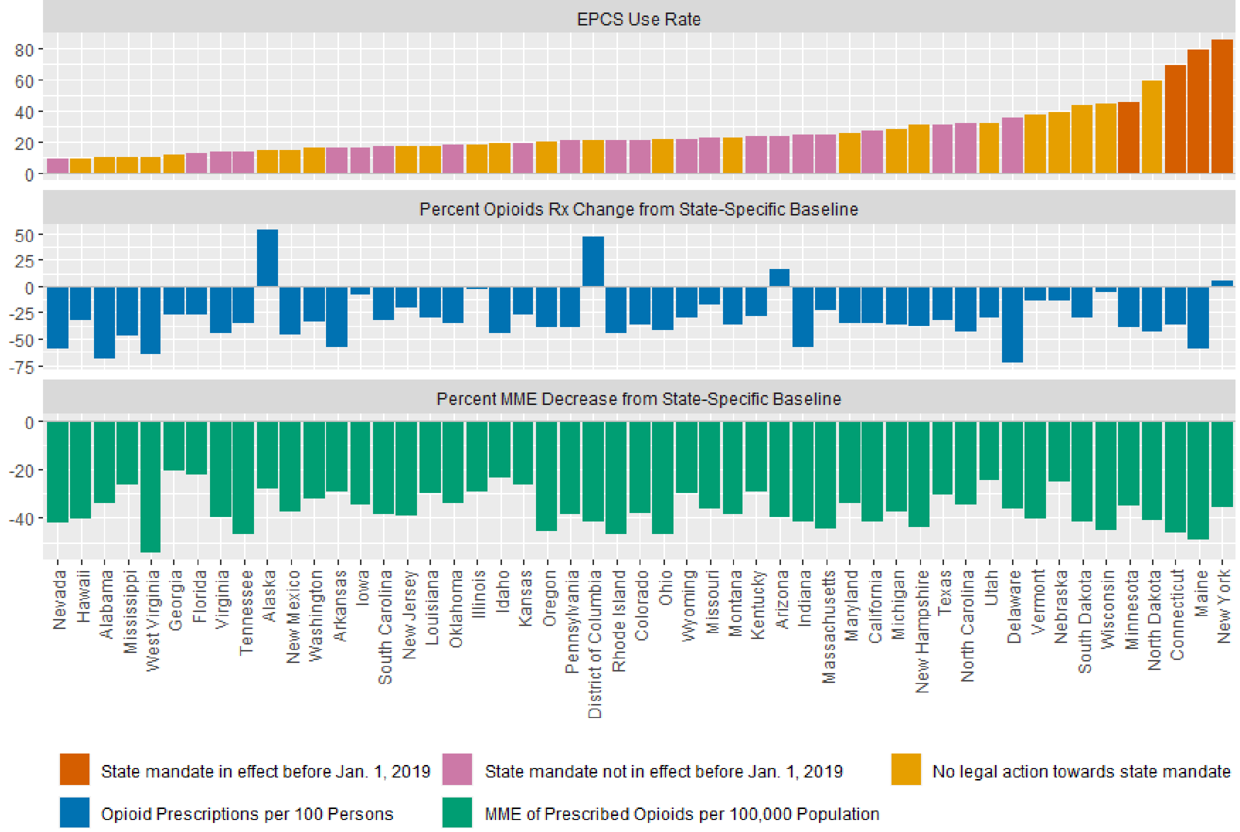

Data on EPCS Use is from annual reports published by SureScripts. Data on opioid prescriptions per 100 persons is from CDC Opioid Prescribing Rate Maps and data on MME of opioids is from U.S. Drug Enforcement Administration's ARCOS.

© 2020 Everson J et al. *JAMA Network Open.*

eTable 1. Multivariable analysis of relationship between EPCS use and prescription opioids relative to state baseline.

	Opioid Prescriptions per 100 persons (Relative to State Level in 2013) (Standard Errors)	Percent MME per 100,000 (Relative to State Level in 2013) (Standard Errors)
EPCS Use Rate (10 percent increments)	1.404**	-0.245
	(0.425)	(0.307)
Unemployment Rate	0.814	4.391
	(0.926)	(2.420)
Poverty Rate	-1.141	-2.893*
	(1.227)	(1.268)
PDMP Implemented	-0.258	-12.408
	(2.367)	(10.064)
Pain Management Clinic Law	3.015	4.015
	(4.246)	(6.988)
Direct Dispensing Law	0.327	0.452
	(1.222)	(1.911)
ED Prescribing Guidelines	-3.628*	-1.279
	(1.702)	(2.068)
Constant	12.847	7.123
	(14.152)	(20.776)
Year Fixed Effects	Included	Included
State Fixed Effects	Included	Included
Observations	459	459
R-squared	0.811	0.730
Number of States	51	51

* p<0.05; ** p<0.01, generated using robust standard errors. EPCS is electronic prescribing of controlled substances. MME is milligram morphine equivalent. PDMP is prescription drug monitoring program. ED is Emergency Department.

eTable 2. Multivariable analysis of relationship between EPCS use and milligram morphine equivalent (MME) per prescription.

	MME per Prescription (Standard Errors	Percent MME per Prescription (Relative to State Level in 2013) (Standard Errors)
EPCS Use Rate (10 percent increments)	-19.208**	-0.020**
	(3.784)	(0.005)
Unemployment Rate	27.171	0.033
	(18.434)	(0.021)
Poverty Rate	-6.168	-0.013
	(13.413)	(0.016)
PDMP Implemented	-99.753	-0.109
	(66.933)	(0.072)
Pain Management Clinic Law	5.085	0.018
	(66.292)	(0.072)
Direct Dispensing Law	-6.953	-0.006
	(17.300)	(0.018)
ED Prescribing Guidelines	22.240	0.027
	(22.489)	(0.028)
Constant	689.538**	-0.113
	(191.813)	(0.220)
Year Fixed Effects	Included	Included
State Fixed Effects	Included	Included
Observations	459	459
R-squared	0.138	0.122
Number of States	51	51

* p<0.05; ** p<0.01, generated using robust standard errors. EPCS is electronic prescribing of controlled substances. MME is milligram morphine equivalent. PDMP is prescription drug monitoring program. ED is Emergency Department.

© 2020 Everson J et al. *JAMA Network Open.*

eTable 3. Multivariable analysis of relationship of interaction between EPCS use, mandated PDMP use and opioid prescriptions.

	Opioid Prescriptions per 100 Persons (Standard Errors)	Percent MME Per 100 Persons (Relative to 2013) (Standard Errors)
EPCS Use Rate (10 percent increments)	2.429*	4.367*
	(0.968)	(1.708)
Mandated PDMP Use	-1.431	4.610
	(1.939)	(3.363)
EPCS Use Rate*PDMP_Mandate	-0.384	-3.768*
	(0.914)	(1.623)
Unemployment Rate	(6.590)	(10.584)
	0.361	4.711
Poverty Rate	(0.771)	(2.482)
	-1.510	-3.465*
PDMP Implemented	(1.218)	(1.591)
	1.500	-11.853
Pain Management Clinic Law	(2.274)	(10.512)
	-2.001	1.027
Direct Dispensing Law	(4.522)	(7.257)
	0.692	1.003
ED Prescribing Guidelines	(1.329)	(2.127)
	-1.748	0.710
Constant	(2.335)	(3.262)
	100.310**	106.234**
Year Fixed Effects	Included	Included
State Fixed Effects	Included	Included
Observations	459	459
R-squared	0.737	0.687
Number of States	51	51

* p<0.05; ** p<0.01, generated using robust standard errors. EPCS is electronic prescribing of controlled substances. MME is milligram morphine equivalent. PDMP is prescription drug monitoring program. ED is Emergency Department.

© 2020 Everson J et al. *JAMA Network Open.*
